# Recent advances in anesthesia of the obese patient

**DOI:** 10.12688/f1000research.15093.1

**Published:** 2018-08-06

**Authors:** Jay B. Brodsky

**Affiliations:** 1 Department of Anesthesiology, Perioperative and Pain Medicine, Stanford University Medical Center, Stanford, USA

**Keywords:** Safe Apnea Period, Propofol, Sugammadex

## Abstract

The anesthetic management of an obese patient can be challenging because of the altered anatomy and physiology associated with obesity. In this article, I review the recent medical literature and highlight some of the controversies in the airway management and drug dosing of morbidly obese patients.

## Introduction

The alarming worldwide increase in obesity, particularly extreme or “morbid obesity” (defined as a body mass index [BMI] of more than 40 kg/m ^2^), means that all healthcare providers must be familiar with the important anatomic and physiologic changes that are unique to these patients. This is especially true for anesthesiologists, since the obese patient can present challenges in many areas of their perioperative management.

## Airway management

A “difficult airway” has been defined as the situation where a trained anesthesiologist experiences difficulty with bag-mask ventilation and/or tracheal intubation ^1^. Difficulty with face mask ventilation is frequently encountered in morbidly obese patients, but whether obesity by itself is a risk factor for a difficult tracheal intubation still remains controversial.

At higher body weight, the odds of a difficult tracheal intubation, which was defined as requiring more than one attempt with direct laryngoscopy (DL), were greater in obese patients compared to lean patients. However, there was no increase in the number of intubation attempts as BMI increased: by this definition, difficult intubation was no more likely in a morbidly obese patient than in a patient who was only moderately obese ^2^. This study, like many previous reports, found that obesity may be associated with a greater risk of a difficult tracheal intubation, but more obesity doesn’t convey more risk.

It is still unclear whether video-assisted laryngoscopy (VAL) rather than conventional DL should be the routine approach to tracheal intubation for obese patients. Many believe that VAL reduces the number of failed intubation attempts by improving the glottic view while also reducing laryngeal/airway trauma. However, a recent meta-analysis failed to demonstrate that VAL, when compared to conventional DL, actually decreased the number of intubation attempts or the incidence of hypoxia or respiratory complications. In addition, VAL did not shorten the time required for successful intubation in adult subjects ^3^.

Airway management remains a challenge for all obese patients because their safe apnea period (SAP), that is the time between muscle paralysis and apnea until oxyhemoglobin saturation (SpO _2_) drops to potentially dangerous levels, is extremely short (SAP of 2–3 minutes) compared to normal-weight patients (SAP of 8–10 minutes). Therefore, an airway must be secured rapidly and/or the duration of SAP must be increased to allow additional time for successful intubation of the trachea.

Recently, there has been increased interest in apneic or “diffusion” oxygenation to increase SAP. During apnea, the body continues to consume approximately 250 mL/minute of oxygen (O _2_) while producing approximately 200 mL/minute of carbon dioxide (CO _2_). Most of the CO _2_ remains in the blood, resulting in a progressive respiratory acidosis, but the O _2_ is consumed from the alveoli with a loss of lung volume. The resulting pressure differential causes mass movement of gas from the upper airways into the alveoli. Therefore, delivery of a high concentration of O _2_ at any airway level (pharynx, trachea, alveoli) can increase alveolar O _2_ stores and delay or prevent desaturation.

Apnea time of patients with difficult airways, including several obese patients undergoing general anesthesia for upper airway operations, was extended with continuous delivery of O _2_ by “transnasal humidified rapid-insufflation ventilatory exchange” (THRIVE). THRIVE combines the benefits of apneic oxygenation with continuous positive airway pressure (CPAP) and gas exchange through flow-dependent dead-space flushing. THRIVE using 70 L/minute of humidified O _2_ was begun as an adjunct to routine pre-oxygenation and then continued both during intravenous induction of anesthesia and following paralysis. Apnea time was measured from the initiation of neuromuscular blockade until jet ventilation or positive-pressure ventilation was instituted and/or resumption of spontaneous ventilation occurred. During apnea, upper airway patency was maintained with jaw-thrust. The median apnea time for all patients in this study was 14 minutes, and no patient experienced arterial desaturation (SpO _2_ of less than 90%) ^4^.

A study compared arterial oxygenation during apnea following different pre-oxygenation techniques. One group was pre-oxygenated by face mask with 100% O _2_ at a rate of 6 L/minute for 3 minutes with CPAP (15 cm H _2_O), while another group received THRIVE at 30 L/minute for 3 minutes. Following paralysis, “apneic” ventilation continued with 10 L O _2_/minute with CPAP at 15 cm H _2_O in the first group. The THRIVE group received 60 L O _2_/minute. The endpoint was desaturation to an SpO _2_ of less than 90% and/or a maximum apnea time of 12 minutes without desaturation. Both treatments were equally effective in prolonging SAP without desaturation for up to 12 minutes. The CPAP group had an added advantage of lower PaCO _2_ with less acidosis ^5^. The use of high-flow nasal O _2_ during pre-oxygenation and then continued during apnea can also prevent hypoxia before and during intubation attempts by extending SAP ^6^.

In another study, obese patients (BMI 30–40 kg/m ^2^) were randomly assigned to routine pre-oxygenation or pre-oxygenation plus “buccal” oxygenation. Buccal O _2_ was administered via a modified 3.5 mm Ring-Adair-Elwyn (RAE) tracheal tube placed inside the patient’s cheek. Patients receiving buccal oxygenation were much less likely to exhibit an SpO _2_ of less than 95% during 750 seconds of apnea. Median apnea times with an SpO _2_ of 95% or more were prolonged in the buccal oxygenation group compared to the non-buccal oxygenation group ^7^. Thus, clinically important prolongation of SAP in obese patients can be achieved by delivering buccal O _2_ during and after the induction of anesthesia. This approach to apneic oxygenation via an oral route requires lower O _2_ flows and less equipment than THRIVE while improving management of the difficult airway and prolonging SAP.

Prior to anesthetic induction of the morbidly obese patient, the upper body and head should be ramped (“head elevated laryngoscopy position”) with the operating room table in the reverse Trendelenburg position. Pre-oxygenation by face mask should be instituted, but, rather than normal tidal volume inspiration, the morbidly obese patient should be instructed to take deep vital capacity breaths. Some form of apneic oxygenation should also be considered to extend SAP. It is important to emphasize that apneic oxygenation by any route cannot compensate for ineffective pre-oxygenation, and the patient’s upper airway must remain patent for O _2_ to be delivered to the lungs. A non-patent airway results in alveolar collapse and rapid desaturation. Therefore, for successful apneic oxygenation, head elevation, jaw thrust, nasal prongs, or an oral airway may be required.

## Drug dosing

Obesity has significant effects on the metabolism and pharmacokinetic profiles of most anesthetic agents. Propofol is the drug most frequently used for the induction of general anesthesia, but the appropriate dosing in obese patients remains controversial. Although bolus dose recommendations based on actual or total body weight (TBW) are valid in normal-weight patients, large doses based on TBW in morbidly obese patients can be dangerous. A prospective study randomized morbidly obese subjects (BMI 40 kg/m ^2^ or more) to receive a propofol infusion (100 mg/kg/hour) for induction of anesthesia based on their TBW or lean body weight (LBW). Control subjects (BMI 25 kg/m ^2^ or less) also received a propofol infusion (100 mg/kg/hour) based on actual (TBW) weight. All subjects were given a 20 mL syringe filled with saline to hold between the thumb and index finger of the hand opposite the intravenous drip and were told to not drop it. During propofol infusion, the moment the syringe was dropped was used as the marker for loss of consciousness (LOC), at which point the propofol infusion was discontinued. The total propofol dose (mg/kg) required for syringe drop and the time to LOC were similar between control normal-weight subjects and morbidly obese subjects given propofol based on LBW. Morbidly obese subjects receiving propofol based on TBW received significantly larger propofol doses and had significantly shorter times to LOC ^8^. These findings suggested that LBW is the appropriate dosing scalar for propofol to achieve LOC at induction of anesthesia in morbidly obese patients.

A more recent study challenged these recommendations. Morbidly obese patients (BMI 40 kg/m ^2^ or more) were randomized to either a non-scalar method utilizing a brain function monitoring device (bispectral index [BIS]) or a scalar method based on LBW. Anesthesia was induced with either a propofol infusion of 100 mg/kg/hour to an initial target end-point (BIS = 50) or until a pre-calculated dose of 2.6 mg/kg based on calculated LBW was administered. Induction was assessed using the Observer’s Assessment Alertness/Sedation (OAA/S) scale, where a lack of response to a painful trapezius squeeze signified a score of 0. If an OAA/S score of 0 was not achieved, the propofol infusion was continued until the OAA/S score reached 0. The induction dose of propofol based on the BIS index end-point was different from the induction dose based on calculated LBW. The majority of the patients in the LBW group required additional propofol to achieve an OAA/S score of 0. Based on these findings, the authors questioned how useful LBW is as a scalar to accurately estimate the correct dose of propofol to be administered to morbidly obese patients ^9^.

The apparent contradiction between propofol dosing recommendations for morbidly obese patients was discussed in an editorial ^10^. The differences in study end-points ******** was because significantly different levels of anesthesia were attained in the two studies. Levels of sedation range from minimal and moderate sedation to deep sedation and finally to general anesthesia. In the first study, LOC was defined as when the patient dropped a syringe at initial moment of relaxation ^7^. This level of LOC resembles moderate or deep sedation corresponding to an OAA/S score of 2 or lower. In contrast, in the second study, the lack of response to a painful stimulus with an OAA/S score of 0 was chosen for LOC, a level that corresponds with a much deeper sedation level consistent with general anesthesia ^8^.

Since the administration of high-dose propofol can have significant hemodynamic consequences, we recommend LBW for propofol dosing for induction of anesthesia in morbidly obese patients, especially when a “balanced induction” using an opioid to supplement the propofol is also used.

Intravenous propofol is an effective method of sedation for patients undergoing procedures requiring sedation outside the operating room. Its use by non-anesthesia professionals has been advocated. A retrospective cohort study compared propofol sedation of obese patients (BMI 34–80 kg/m ^2^) undergoing pre-bariatric surgical outpatient esophagogastroduodenoscopy (EGD) with non-obese control patients (BMI 25 kg/m ^2^ or less) undergoing diagnostic EGD. The obese group had a high incidence of sleep apnea (62 versus 8%; *P* <0.001), experienced more SpO _2_ desaturations (22 versus 7%; *P* <0.001), and received more chin lift maneuvers (20 versus 6%; *P* <0.001) than did the lean group. Yet, despite these differences, the authors concluded that with appropriate training of endoscopy personnel, propofol sedation without anesthesia personnel being present was still a safe method of sedation in severely obese patients undergoing outpatient upper endoscopy ^11^. The high incidence of “desaturation” and the frequent need for chin lift presumably to open the patients’ upper airways suggest a different conclusion: the potential for serious airway complications is always present with propofol sedation, and anesthesia personnel should be managing these high-risk obese patients.

Since its US Food and Drug Administration approval in 2015, sugammadex has replaced neostigmine as the drug of choice for the reversal of neuromuscular blockade by steroidal muscle relaxants (rocuronium, vecuronium). The manufacturer recommends that the dose of sugammadex be based on actual body weight and the level of neuromuscular blockade.

A meta-analysis of 27 trials evaluated recovery times following sugammadex reversal in patients with BMIs of 30 kg/m ^2^ or more (obese) and less than 30 kg/m ^2^ (non-obese). Sugammadex was administered based on actual weight. No clinically relevant correlation was observed between BMI and time to recovery. At high doses of sugammadex based on TBW, there was rapid recovery from neuromuscular blockade in both obese and non-obese patients ^12^.

For moderate neuromuscular blockade, defined as recovery of two or more twitches in response to train-of-four stimulation, a dose of 2 mg/kg TBW is recommended by the manufacturer, irrespective of actual weight. Several studies have demonstrated that this amount is both unnecessary and expensive in morbidly obese patients.

Bariatric surgical patients received sugammadex based on either TBW or ideal body weight (IBW). The mean dose of sugammadex in the IBW group was 4 mg/kg based on IBW plus 35%. Time to complete reversal of neuromuscular blockade was not significantly different between the two groups, even though sugammadex doses were significantly lower in the IBW group ^13^.

Even lower doses of sugammadex have been used successfully in morbidly obese patients. A dose of sugammadex of 4 mg/kg based on IBW only allowed suitable reversal of deep rocuronium-induced neuromuscular blockade ^14^. A single vial of sugammadex contains 200 mg of the drug, which is considered adequate for the reversal of neuromuscular blockade for obese patients. Since morbidly obese patients are at risk for hypoventilation and upper airway obstruction following tracheal extubation, neuromuscular blockade monitoring remains essential to detect residual neuromuscular blockade or recurarization.

## Conclusion

The anesthetic management of the morbidly obese patient continues to evolve and change, and many controversies remain. Techniques that may work well for a normal-weight patient may be inappropriate and even unsafe for an obese patient. Published studies, based on the experiences of others, help to guide anesthesiologists in the care of these patients.
